# Declining melanoma incidence among younger adults in Sweden: insights from histopathological subtypes and the role of immigration

**DOI:** 10.2340/1651-226X.2026.45887

**Published:** 2026-07-30

**Authors:** Yodit Girmay, Rasmus Mikiver, Jan Lapins, Karolin Isaksson, Hildur Helgadottir

**Affiliations:** a Department of Oncology and Pathology, Karolinska Institutet, Stockholm, Sweden; b Department of Biomedical and Clinical Sciences, Linköping University, Linköping, Sweden; c Regional Cancer Center South East Sweden, Linköping, Sweden; d Department of Dermatology, Karolinska University Hospital, Stockholm, Sweden; e Dermatology and Venereology Unit, Department of Medicine, Karolinska Institutet, Stockholm, Sweden; f Department of Clinical Sciences, Surgery, Lund University, Lund, Sweden; g Department of Surgery, Skåne University Hospital, Kristianstad, Sweden; h Theme Cancer, Karolinska University Hospital, Stockholm, Sweden

**Keywords:** Cutaneous melanoma, ultraviolet radiation, acral melanoma, spitz melanoma, superficial spreading melanoma, histopathological subtypes

## Introduction

Since the 1950s, the incidence of melanoma has steadily increased among fair-skinned populations worldwide [1]. While the most common melanoma subtypes are ultraviolet radiation (UV) associated, other less common histopathological subtypes develop independently of UV exposure. These subtypes differ in their biological behaviour, association with UV exposure, and prognostic outcomes. Superficial spreading melanoma (SSM) is linked to intermittent UV exposure, lentigo maligna melanoma (LMM) to chronic sun damage, nodular melanoma (NM) has a mixed UV association, while acral lentiginous melanoma (ALM) and Spitz melanoma are largely UV-independent [2].

Sweden has the sixth highest incidence of melanoma worldwide [3]. However, a recent study reported a decline in melanoma incidence among younger adults in Sweden starting in 2015, marking the first indication of a trend reversal in Europe after decades of increasing rates [4]. Proposed explanations include UV-protection campaigns [5]. Another theory is that the increased immigration of populations with darker skin tones may have contributed to this trend shift as foreign-born individuals now comprise about 20% of Sweden’s population, including 11% born outside Europe [6].

This study further examined the recent decline in melanoma incidence among younger adults in Sweden by adding 2 more years compared with our initial report [4], assessing subtype-specific trends, and evaluating whether immigration contributed to the observed decrease.

## Methods

This study included all individuals aged 20–59 years in Sweden diagnosed with histologically confirmed primary invasive melanoma between 1998 and 2024. Based on our previous report identifying an incidence shift at age 50 years [4], patients were categorised as 20–49 years or 50–59 years. Further details are provided in the Supplementary Methods.

## Results

In the years 1998–2024, 17,109 melanomas were diagnosed in 16,426 patients aged 20–49 years and 14,368 melanomas in 14,998 patients aged 50–59 years (Supplementary Table S1).

### Melanoma incidence in the whole population

Among individuals aged 20–49 years, the incidence increased steadily until 2014, peaking at 15.4 and 27.7, and then declining to 14.5 and 22.9 cases per 100,000 in males and females, respectively (Table 1 and Supplementary Figure S1). In contrast, among the 50–59-year group, the incidence continued to increase over nearly the entire period in both sexes.

**Table 1:** Annual percentage change in primary invasive cutaneous melanoma incidence in Sweden by age group and melanoma subtypes, from 1998 to 2024.

Category	Age group (years)	Males Year segment APC (LCL to UCL) *p*-value		Females Year segment APC (LCL to UCL) *p*-value	
		Segment 1	Segment 2	Segment 1	Segment 2
All melanomas	20–49	2000–2014	2014–2024	2000–2014	2014–2024
		3.9 (3.1 to 4.7)	–1.1 (–2.2 to 0.0)	5.1 (4.3 to 5.9)	–1.9 (–2.9 to –0.9)
		*p* < 0.001	*p* = 0.054	*p* < 0.001	*p* < 0.001
	50–59	2000–2024	–	2000–2004	2004–2024
		4.3 (3.9 to 4.8)		–0.7 (–7.2 to 6.3)	4.8 (4.3 to 5.3)
		*p* < 0.001		*p* = 0.829	*p* < 0.001
Superficial spreading melanoma	20–49	1998–2015	2015–2024	1998–2014	2014–2024
		3.9 (3.0 to 4.7)	0.4 (–1.3 to 2.1)	5.2 (4.6 to 5.8)	–0.7 (–1.6 to 0.3)
		*p* < 0.001	*p* = 0.656	*p* < 0.001	*p* = 0.161
	50–59	1998–2006	2006–2024	1998–2004	2004–2024
		2.5 (–0.9 to 6.1)	6.0 (5.2 to 6.7)	–1.1 (–5.5 to 3.6)	5.9 (5.3 to 6.5)
		*p* = 0.146	*p* < 0.001	*p* = 0.632	*p* < 0.001
Nodular melanoma	20–49	1998–2010	2010–2024	1998–2024	–
		2.8 (0.0 to 5.7)	–6.8 (–9.0 to –4.4)	–1.8 (–3.1 to –0.5)	
		*p* = 0.047	*p* < 0.001	*p* = 0.010	
	50–59	1998–2024	–	1998–2024	–
		0.3 (–0.7 to 1.4)		1.3 (0.5 to 2.1)	
		*p* = 0.502		*p* = 0.002	
Lentigo maligna melanoma	20–49	1998–2013	2013–2024	1998–2024	–
		8.7 (–2.9 to 21.7)	–10.4 (–27.2 to 10.2)	1.4 (–1.2 to 4.1)	
		*p* = 0.110	*p* = 0.216	*p* = 0.239	
	50–59	1998–2024	–	1998–2024	–
		3.2 (0.5 to 5.9)		3.2 (1.2 to 5.2)	
		*p* = 0.025		*p* = 0.006	
Acral lentiginous melanoma	20–49	1998–2024	–	1998–2024	–
		–3.7 (–10.5 to 3.6)		1.6 (–1.1 to 4.3)	
		*p* = 0.261		*p* = 0.209	
	50–59	1998–2024	–	1998–2024	–
		0.5 (–3.0 to 4.1)		0.9 (–2.2 to 4.1)	
		*p* = 0.751		*p* = 0.508	
Spitz* melanoma	20–49	2009–2024	–	2009–2024	–
		–4.9 (–11.4 to 2.1)		–3.0 (–8.8 to 3.2)	
		*p* = 0.134		*p* = 0.280	
	50–59	2009–2024	–	2009–2024	–
		–0.1 (–10.3 to 11.3)		–0.3 (–8.2 to 8.2)	
		*p* = 0.988		*p* = 0.927	

LCL: Lower Confidence Limit, UCL: Upper Confidence Limit; APC: annual percentage change.

* Data on Spitz melanomas was only available from 2009.

### Superficial spreading melanoma

The SSMs that constituted the majority of all tumours also had incidence trends similar to those observed for the cohort of all melanomas (Table 1 and Supplementary Figure S2). From 1998 until 2014 in males and until 2015 in females, there were statistically significant yearly increases in the SSMs in those aged 20–49 years. In 2014, the yearly incidence of SSM was 11.3 and 20.3 cases per 100,000 in males and females, respectively. In 2015–2024, the yearly incidence increase of SSM declined to 0.4% (*p* = 0.656) in males, while there was a yearly decline of –0.7% in females (*p* = 0.161) between 2014 and 2024. Similarly, for the whole population, among the 50–59-year group, the incidence continued to statistically increase over nearly the entire period, with an incidence reaching 51.0 and 65.6 cases per 100,000 in males and females, respectively, in 2024.

### Nodular melanoma

The trends for NM were somewhat different from those of the entire population and SSMs (Table 1 and Supplementary Figure S3). In the 20–49-year-old males there was a statistically significant increase until 2010 (peaking at 1.9 cases per 100,000), followed by a statistically significant annual decrease until 2024. In the 20–49-year-old females, there was no such peak, with the highest incidence of 2.2 cases per 100,000 in 1998 and then a consistent, statistically significant yearly decrease during the whole period until 2024. In the 50–59-year-olds, there was a gradual increase in NM during the entire period, with a yearly increase of 0.3% in males (*p* = 0.502) and 1.3% in females (*p* = 0.002), and an incidence reaching 5.0 cases per 100,000 in both males and females, respectively, in 2024.

### Lentigo maligna melanoma

The incidence of LMMs remained low in the 20–49-year-old population, in the males peaking at 0.3 per 100,000 in 2013–2015, and in the females at 0.4 cases per 100,000 in 2022–2024 (Table 1 and Supplementary Figure S4). In both male and female 50–59-year-olds, however, there was a statistically significant incidence increase in both sexes throughout the study period, reaching 1.9 and 2.4 cases per 100,000 in 2024.

### Spitz melanoma

The incidence of Spitz melanoma remained low, well under 1 case per 100,000 in all groups (Table 1 and Supplementary Figure S5). However, while the incidence remained stable among 50–59-year-olds in the 2009–2024 period, during which data were available on this subtype, while a non-significant downward trend was observed in 20–49-year-olds for both males (–4.9%) and females (–3.0%) 20–49-year-olds.

### Acral lentiginous melanoma

No consistent or statistically significant temporal changes were detected over the study period for ALM in either the younger or older population, with a maximum incidence per 100,000 well below one case (Table 1 and Supplementary Figure S6).

### The incidence of melanoma, excluding individuals born in Africa or Asia

**Figure 1:**
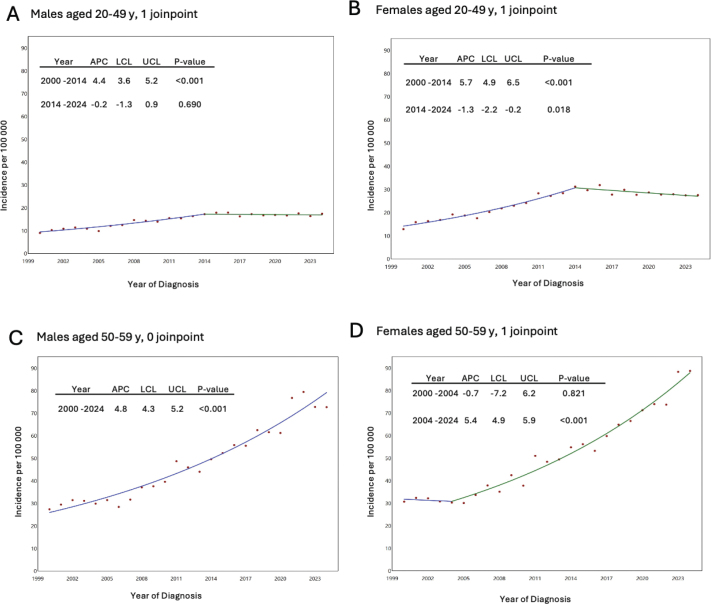
(A–D) Annual percentage change (APC) in primary invasive cutaneous melanoma incidence in Sweden by age group, from 2000 to 2024; Inhabitants born in Africa or Asia excluded. LCL: lower confidence limit; UCL: upper confidence limit.

Excluding inhabitants born in Asia and Africa resulted in a similar pattern among those aged 20–49 years, with a peak in 2014, in both males and females, although the initial increase was slightly steeper, and the subsequent stabilisation was somewhat less pronounced than in the whole population (Figure 1). Until 2014, there was a significant yearly increase in males and females aged 20–49 years, and in 2014–2024 the trend stabilised to –0.2% (*p* = 0.690) in males and decreased –1.3 in females (*p* = 0.018). In the total population, among the 50–59-year group, excluding those born in Africa or Asia, the incidence continued to increase significantly over nearly the entire period, with a significant yearly increase of 4.8% in males (2000–2024) and 5.4% in females (2004–2024) (Figure 1C–D).

## Discussion

In this nationwide study, melanoma incidence continued to decline in younger adults in Sweden. Analyses excluding inhabitants born in low-incidence regions of Africa or Asia, the primary regions of immigration to Sweden in recent decades, showed similar temporal patterns compared with the total population. These findings suggest that recent immigration is unlikely to be the main explanation for the decline, contrary to what has previously been hypothesised in the Australian context [7]. Supporting our findings, a study showed that the reduction in melanoma among young people in Australia was not fully accounted for by immigration alone [8].

Subtype-specific analyses support this interpretation. SSM, a low cumulative sun damage (CSD) subtype linked to intermittent, high-intensity UV exposure [9], largely mirrored the overall melanoma trend, with incidence levelling off among adults aged 20–49 years while continuing to rise among those aged 50–59 years. In contrast, LMM, a high-CSD subtype associated with chronic sun damage and older age [10, 11], continued to increase mainly in the older age group. NM showed a similar age divergence, with more favourable trends among younger adults but continued stability or increase among adults aged 50–59 years, consistent with its more heterogeneous aetiology [12]. UV-independent subtypes, including ALM and Spitz melanoma [13–18], remained rare and largely stable over time.

In women and men aged 20–49 years, melanoma incidence increased up to around 2014 and then declined. In contrast, in women and men aged 50–59 years (an age group just below the national median age at melanoma diagnosis), the incidence continued to rise without evidence of a recent downturn, consistent with the broader population trend. A plausible explanation for the observed shifts in melanoma incidence is population-level behavioural changes driven by earlier public health campaigns. Beginning in the 1980s–1990s, a series of prevention efforts and normative shifts likely changed UV‐exposure behaviours in ways that are consistent with the later stabilisation or decline in melanoma incidence among adolescents and young adults observed in several Western countries [19–21]. Australia’s long-running Slip! Slop! Slap! and SunSmart programmes normalised sun avoidance at peak UV levels, protective clothing/hat, and sunscreen use [22]. Multiple analyses attribute the downturn in invasive melanoma among younger Australians to these sustained population-wide primary prevention efforts [20].

Sweden’s first comparable national UV-protection campaigns were launched in 1995 by the Swedish Cancer Society and the National Radiation Safety Authority, ‘Sola sakta’ (caution in the sun), with messaging that emphasised protecting children from sunburn, hats/clothing, shade at midday, and sensible sunscreen use [4]. Evidence from Swedish studies supports a change in sun-protection behaviour. Between 2002 and 2007, parents reported greater use of sunscreen, protective clothing and shade, as well as increased avoidance of peak UV hours among 7-year-old children [23]. These changes were accompanied by a marked decline in melanocytic-naevus density. Follow-up data through 2012 showed that improved sun-protection practices persisted and were associated with further reductions in naevus density among older children [24]. Another measurable shift in the 1990s was a marked decrease in the availability of indoor tanning among Swedish adolescents. Over the past two to three decades, legislative measures and regulatory advisories from Sweden’s National Radiation Safety Authority have progressively limited access to sunbeds [25] and surveys in Stockholm County have documented steep declines in their use between 1993 and 1999 [26]. The potential importance of this behavioural change is supported by a recent analysis of nearly 35,000 participants in the Stockholm Public Health Cohort. In that study, sunbed use more than 10 times before age 30 was associated with higher odds of melanoma and other skin cancers compared with never use, after adjustment for a range of behavioural, social, hereditary, and medical factors [27]. Because indoor tanning delivers intermittent, high-intensity UV exposure, this contraction in the mid-to-late 1990s would be expected to lower melanoma risk in the cohorts who were adolescents then and are today’s 20–49-year-olds.

Taken together, the decline in melanoma incidence in younger adults can be interpreted as protective behaviour first becoming evident at the population level after approximately 20 years. It is however important to point out that although UV radiation is an established causal factor for melanoma, the proportion of melanomas attributable to UV exposure and the relative importance of different patterns of UV exposure are not clear-cut. Melanoma development and diagnosis reflect complex interactions of environmental and host factors. Therefore, the patterns observed are indicative but cannot be interpreted as direct evidence that changes in UV exposure alone explain the observed incidence trends.

## Conclusion

This nationwide study demonstrates that the recent downward trend in melanoma incidence among younger individuals in Sweden is unlikely to be explained mainly by immigration or changes in population composition. Although this study does not directly test any such associations, the trends observed are more consistent with increased awareness and reduced sun exposure driven by previous public health campaigns. Subtype-specific trends support this interpretation, as declines are most pronounced in UV-associated melanomas, whereas UV-independent melanomas remain stable.

## Supplementary Material



## Data Availability

The data supporting the findings of this study are available from the corresponding author Yodit Girmay upon reasonable request.
