# Supplementary material for: Declining melanoma incidence among younger adults in Sweden: insights from histopathological subtypes and the role of immigration
Source: Acta Oncol. 2026 Jul 30;65:45887. doi: 10.2340/1651-226X.2026.45887 (PMC13430635; doi:10.2340/1651-226X.2026.45887)
Supplement: Supplementary file 1 [file AO-65-45887-s1.pdf]

Supplementary material has been published as submitted. It has not been copyedited, or typeset by Acta Oncologica

Supplementary Material

Supplementary Methods

Supplementary Table S1: Summary of study cohorts

Supplementary Figure S1: Whole cohort trends

Supplementary Figure S2: SSM trends

Supplementary Figure S3: NM trends

Supplementary Figure S4: LMM trends

Supplementary Figure S5: Spitz trends

Supplementary Figure S6: ALM trends

Supplementary Methods

Patients

The Swedish Melanoma Register (SweMR) is a nationwide population-based database that prospectively collects clinicopathological information on invasive cutaneous melanoma. Established in 1990, the SweMR achieved nationwide coverage in 1996 and now provides nearly complete national registration of primary melanoma cases.

This study included all individuals in Sweden aged 20–59 years who were diagnosed with histologically confirmed primary invasive melanoma between 1998 and 2024. The incidence rates were based on the total number of registered primary invasive melanomas, not on individual patients. Accordingly, each primary melanoma was counted as a separate event, including multiple primary melanomas diagnosed in the same patient.

Information was retrieved on each case's registered melanoma histopathological subtype, superficial spreading melanoma (SSM), nodular melanoma (NM), lentigo maligna melanoma (LMM), acral lentiginous melanoma (ALM), or Spitz melanoma. Based on our previous report demonstrating an incidence shift at age 50 years(1) patients were categorised as 20–49 or 50–59 years.

Incidence trend analyses were performed for all melanomas combined, in the whole population, and the population without those born in Africa and Asia was done for the years 2000-2024, as the information on birthplace was available from the year 2000 and onwards. For trends in melanoma subtypes, the period was set from 1998 to 2024 except for Spitz melanoma, where data were only available from 2009 and onwards. The reason why the time interval in the melanoma subtypes started in 1998 (and not in 2000 as for the whole population) was that for LMM and ALM, the cases were so few that it was necessary to report them as 3-year average rates, where the first period included 1998-2000, and ending with 2022-2024. Therefore, all incidence analyses of the different subtypes (except Spitz melanoma) started in 1998. Spitz melanomas were reported at 2-year average rates.

Statistical analysis

Incidence rates per 100,000 inhabitants were calculated for each year and presented as annual rates. As no substantial fluctuation was observed during the study period within different age groups in the 20-59-year-old age group, age-standardization was not performed (2). To investigate the potential influence of immigration on melanoma trends, analyses were conducted for the total Swedish population and for a subpopulation excluding inhabitants born in Asia or Africa (3), the primary regions of immigration in the past decade. This exclusion was based on publicly available demographic data from the Swedish Central Bureau of Statistics', as individuals' race or skin colour is a variable that is not included in Swedish health or population databases. The deduction based on birthplace was only performed for the whole population (inhabitants in the denominator) and not for melanoma cases (in the numerator), as this information was only available for the whole population and not on the level of each melanoma case. Based on the substantially lower melanoma incidence in these continents (4).

1. Helgadottir H, Mikiver R, Schultz K, Nielsen K, Portelli F, Lapins J, et al. Melanoma Incidence and Mortality Trends Among Patients Aged 59 Years or Younger in Sweden. JAMA Dermatol. 2024;160(11):1201-10.
2. Statistics Sweden. Population by Age and Sex. Available from: https://www.statistikdatabasen.scb.se/pxweb/en/ssd/START__BE__BE0101__BE0101A/BefolkningR1860N/.
3. Statistics Sweden. S. International Classifications. 2026. Available from: https://www.scb.se/dokumentation/klassifikationer-och-standarder/internationella-indelningar/.
4. International Observatory. IAfRoCotWHOGC. Global Cancer Observatory. 2026. Available from: https://gco.iarc.who.int/today/en.

Supplementary Table S1. Primary Invasive Cutaneous Melanomas Reported in Patients Younger Than 60 Years, Sweden, 1998 to 2024, by age group, sex and melanoma subtype.

	Age group	
	20-49 y	50-59 y
Total*	17109 (53.3%)	14998 (46.7%)
**Male**	6633 (38.8%)	7328 (48.9%)
Histopathological subtype:		
Superficial Spreading Melanoma	4861 (73.3%)	5222 (71.3%)
Nodular Melanoma	660 (10.0%)	900 (12.3%)
Lentigo Maligna Melanoma	104 (1.6%)	244 (3.3%)
Acral Lentiginous Melanoma	36 (0.5%)	56 (0.8%)
Spitz melanoma**	80 (1.2%)	54 (0.7%)
Other	707 (10.7%)	647 (8.8%)
Non-classifiable	96 (1.5%)	117 (1.6%)
Missing	89 (1.3%)	88 (1.2%)
**Female**	10476 (61.2%)	7670 (51.1%)
Histopathological subtype:		
Superficial Spreading Melanoma	7929 (75,7 %)	5596 (73,0 %)
Nodular Melanoma	757 (7,2%)	760 (9,9%)
Lentigo Maligna Melanoma	142 (1,4%)	231 (3,0%)
Acral Lentiginous Melanoma	83 (0,8%)	760 (9.9%)
Spitz melanoma	161 (1,5 %)	69 (0,9 %)
Other	1077 (10.3%)	722 (9.4%)
Non-classifiable	157 (1.5%)	121 (1.6%)
Missing	170 (1.6%)	90 (1.2%)

*The table is based on 32,107 melanomas diagnosed in 30,551 individuals.
**Data was available from 2009 and onward.

Supplement. Declining Melanoma Incidence among Younger Adults in Sweden: Insights from Histopathological Subtypes and the Role of Immigration

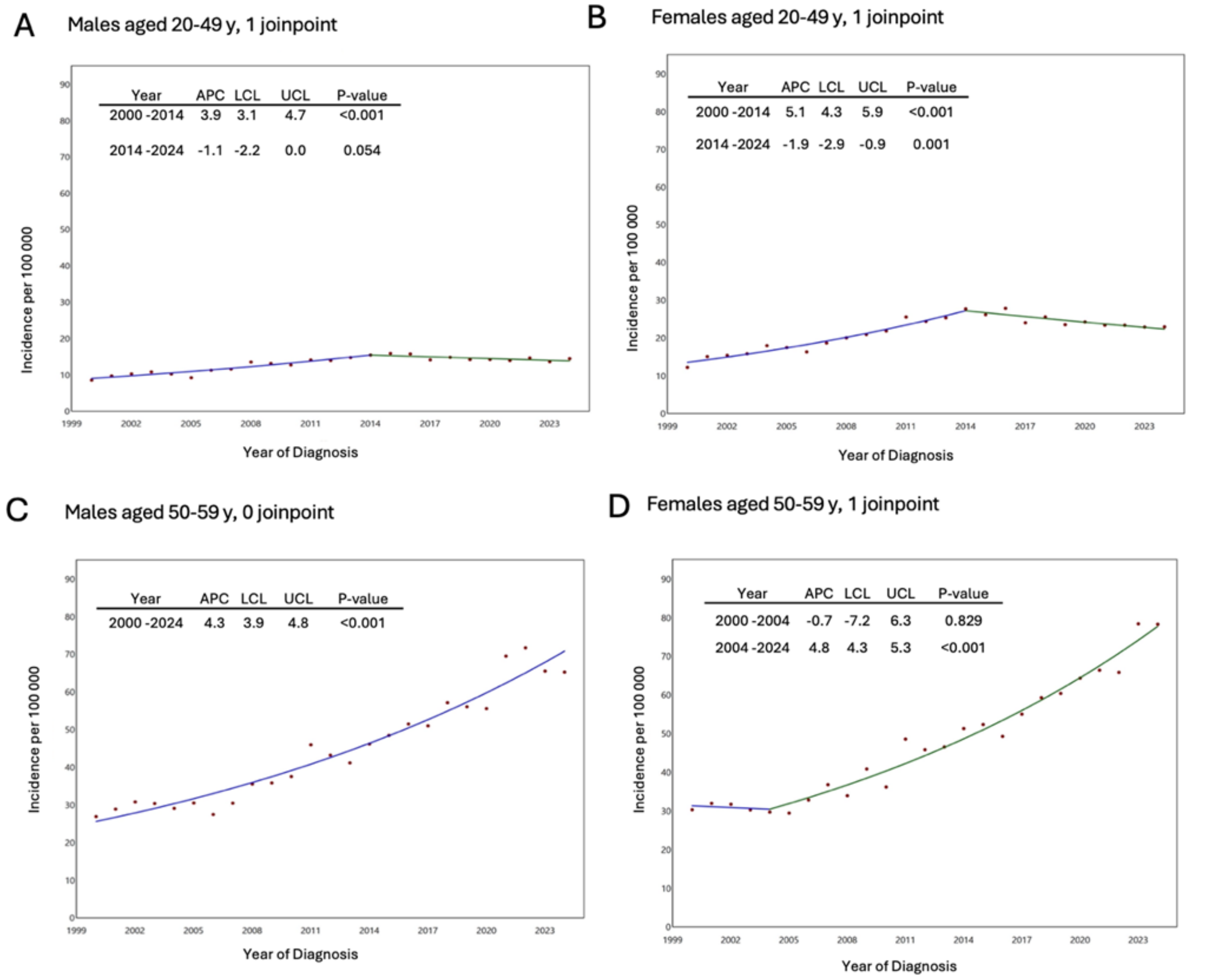

Figure S1A-D. Annual Percentage Change (APC) in the Primary Invasive Cutaneous Melanoma Incidence in Sweden From 2000 to 2024, by Age Group. LCL, lower confidence limit; and UCL, upper confidence limit.

Supplement. Declining Melanoma Incidence among Younger Adults in Sweden: Insights from Histopathological Subtypes and the Role of Immigration

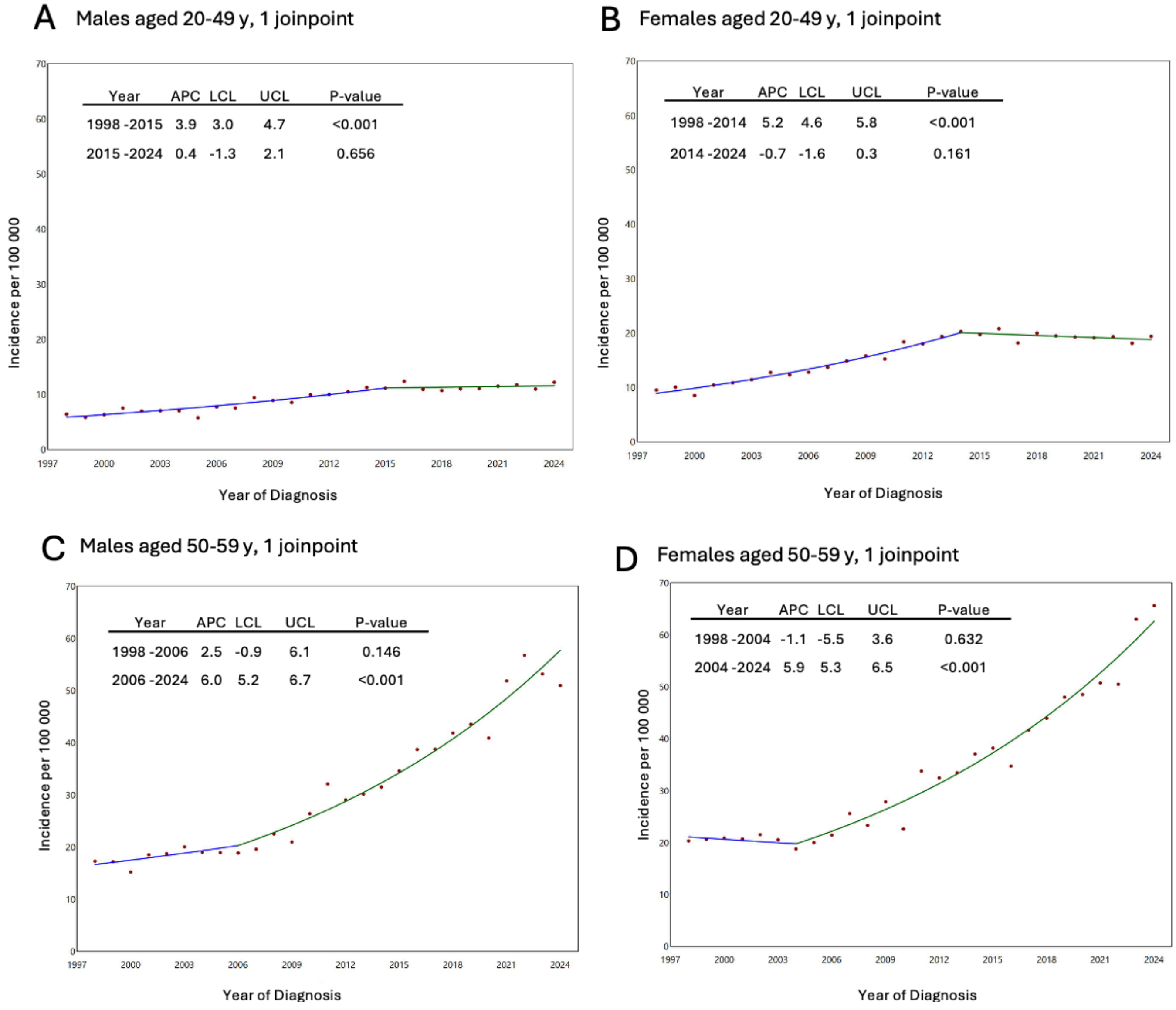

Figure S2A-D. Annual Percentage Change (APC) in Superficial Spreading Melanoma Incidence in Sweden From 1998 to 2024, by Age Group. LCL, lower confidence limit; and UCL, upper confidence limit.

Supplement. Declining Melanoma Incidence among Younger Adults in Sweden:
Insights from Histopathological Subtypes and the Role of Immigration

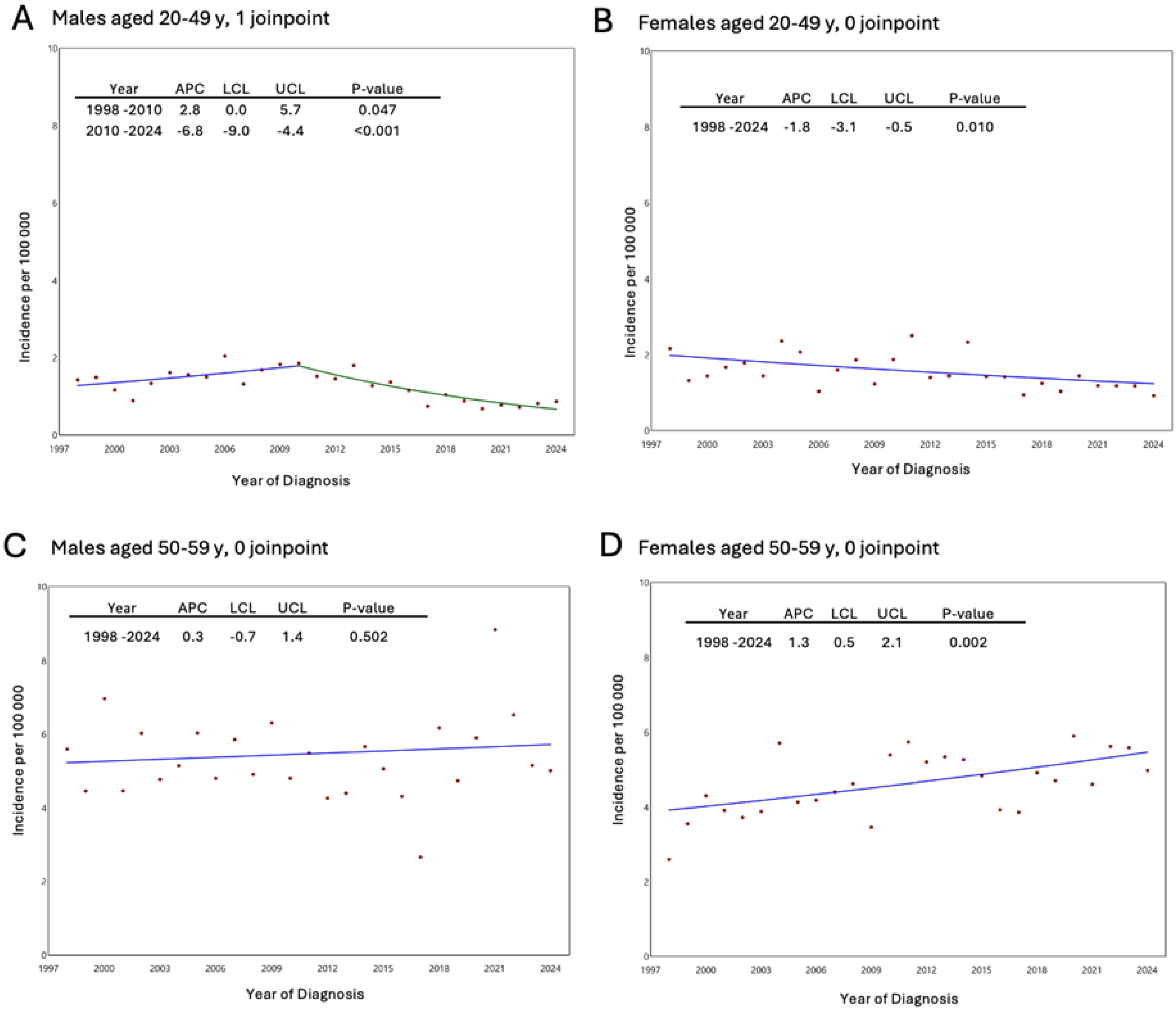

Figure S3A-D. Annual Percentage Change (APC) in Nodular Melanoma Incidence in Sweden From 1998 to 2024, by Age Group. LCL, lower confidence limit; and UCL, upper confidence limit.

Supplement. Declining Melanoma Incidence among Younger Adults in Sweden:
Insights from Histopathological Subtypes and the Role of Immigration

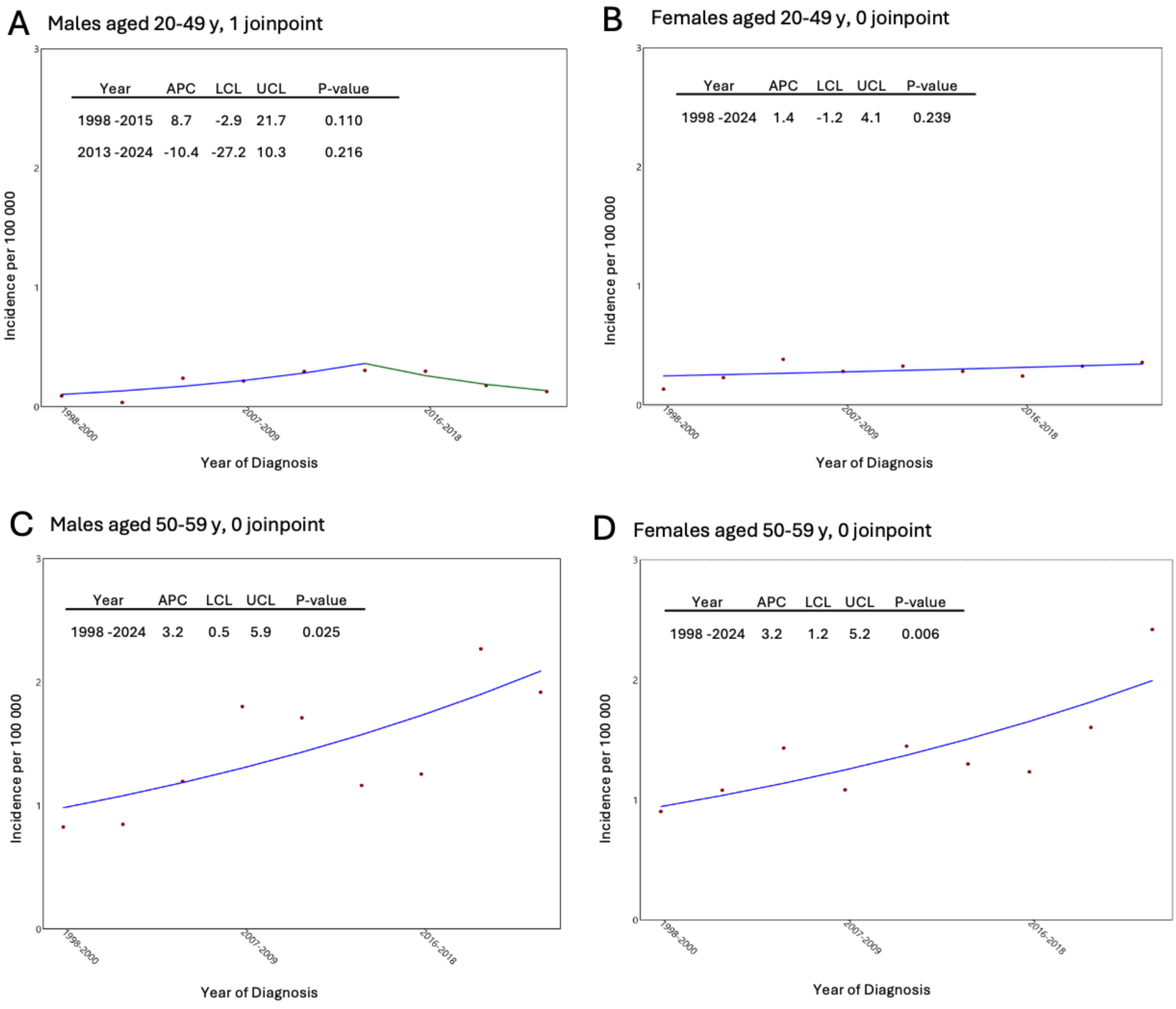

Figure S4A-D. Annual Percentage Change (APC) in Lentigo Maligna Melanoma Incidence in Sweden From 1998 to 2024, by Age Group. LCL, lower confidence limit; and UCL, upper confidence limit.

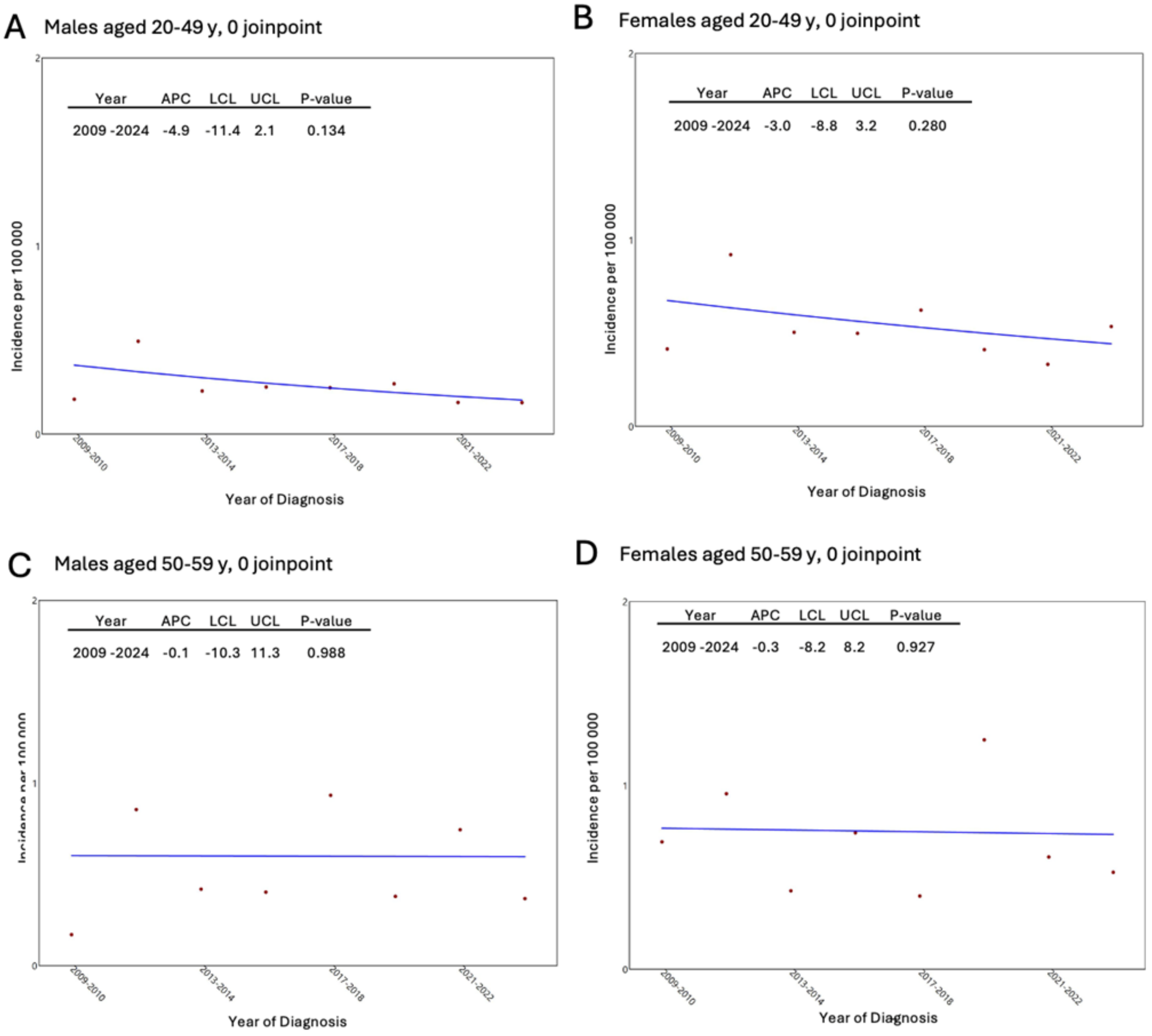

Figure S5A-D. Annual Percentage Change (APC) in Spitz Melanoma Incidence in Sweden From 2009 to 2024, by Age Group. LCL, lower confidence limit; and UCL, upper confidence limit.

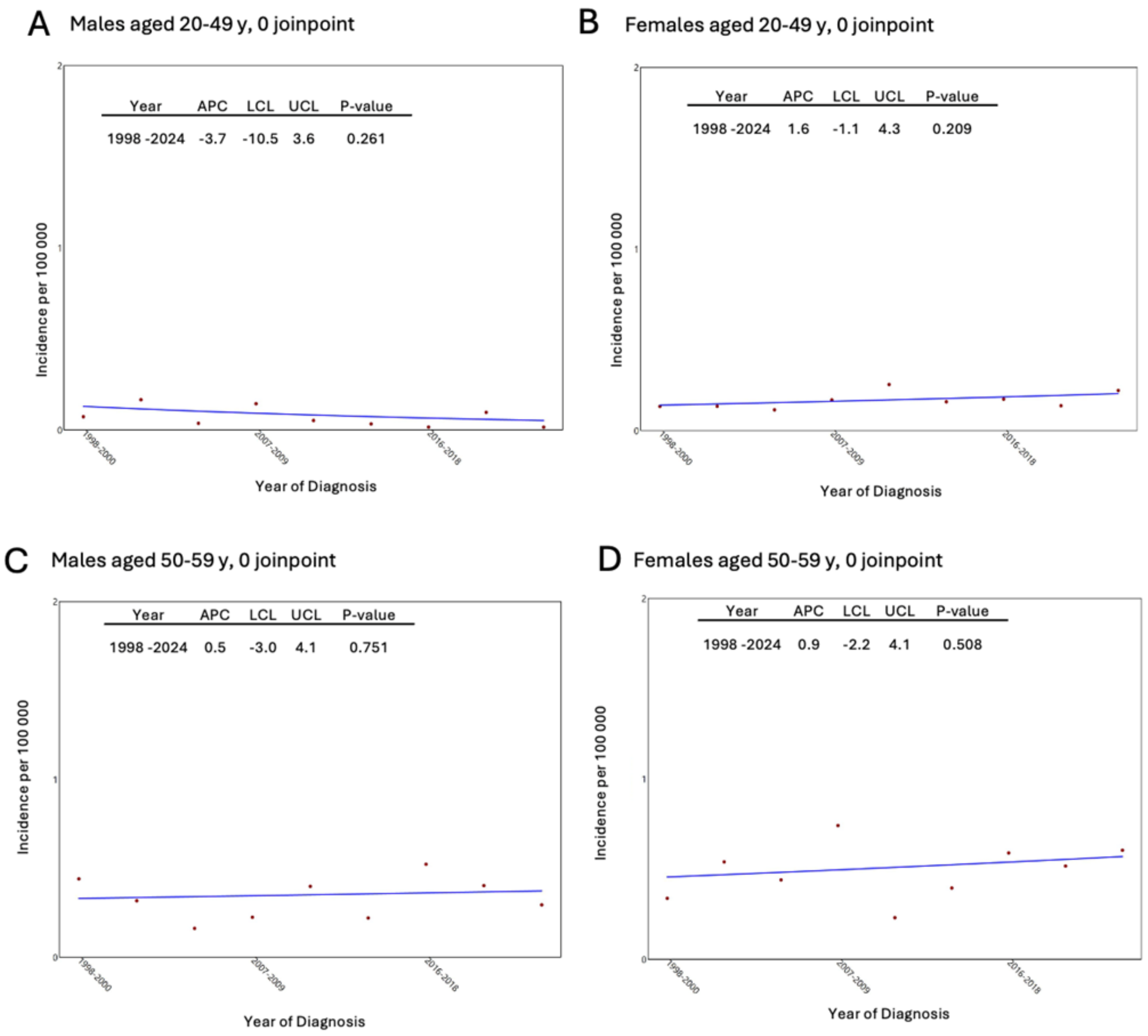

Figure S6A-D. Annual Percentage Change (APC) in Acral Lentiginous Melanoma Incidence in Sweden From 1998 to 2024, by Age Group. LCL, lower confidence limit; and UCL, upper confidence limit.
